# Multilevel evidence of MECP2-associated mitochondrial dysfunction and its therapeutic implications

**DOI:** 10.3389/fpsyt.2023.1301272

**Published:** 2024-01-05

**Authors:** Peter Balicza, Andras Gezsi, Mariann Fedor, Judit C. Sagi, Aniko Gal, Noemi Agnes Varga, Maria Judit Molnar

**Affiliations:** ^1^Institute of Genomic Medicine and Rare Disorders, Semmelweis University, Budapest, Hungary; ^2^Eotvos Lorand Research Network, Multiomic Neurodegeneration Research Group, Budapest, Hungary; ^3^Department of Measurement and Information Systems, Budapest University of Technology and Economics, Budapest, Hungary

**Keywords:** MECP2 mutation, Rett syndrome, learning disability, anxiety, negative symptoms, cariprazine, mitochondrial dysfunction, RNA sequencing

## Abstract

We present a male patient carrying a pathogenic MECP2 p. Arg179Trp variant with predominant negative psychiatric features and multilevel evidence of mitochondrial dysfunction who responded to the cariprazine treatment. He had delayed speech development and later experienced severe social anxiety, learning disabilities, cognitive slowing, and predominant negative psychiatric symptoms associated with rigidity. Clinical examinations showed multisystemic involvement. Together with elevated ergometric lactate levels, the clinical picture suggested mitochondrial disease, which was also supported by muscle histopathology. Exploratory transcriptome analysis also revealed the involvement of metabolic and oxidative phosphorylation pathways. Whole-exome sequencing identified a pathogenic MECP2 variant, which can explain both the dopamine imbalance and mitochondrial dysfunction in this patient. Mitochondrial dysfunction was previously suggested in classical Rett syndrome, and we detected related phenotype evidence on multiple consistent levels for the first time in a MECP2 variant carrier male. This study further supports the importance of the MECP2 gene in the mitochondrial pathways, which can open the gate for more personalized therapeutic interventions. Good cariprazine response highlights the role of dopamine dysfunction in the complex psychiatric symptoms of Rett syndrome. This can help identify the optimal treatment strategy from a transdiagnostic perspective instead of a classical diagnostic category.

## Introduction

The MECP2 gene was classically associated with Rett syndrome in female individuals with presumed lethality in hemizygous male individuals (1). With growing knowledge on the gene's contribution to human diseases, currently, we talk about “MECP2 disorders” (2), with a phenotypic spectrum both in female and male individuals. In male patients, the spectrum involves male Rett syndrome encephalopathy, atypical Rett syndrome, neonatal or progressive encephalopathy, and cognitive impairment, with severely affected male patients tending to harbor female Rett syndrome-causing variants (3). Early infancy is when the first signs of Rett syndrome symptoms start to appear in female patients after a period of typical neurological and physical development. Signs grow gradually across various phases. Loss of acquired speech and motor skills, repetitive hand motions, respiratory issues, and seizures are all possible symptoms. Additionally, rare instances of gastrointestinal problems, hypoplasia of the feet, early-onset osteoporosis, bruxism, and screaming fits may occur in classical Rett syndrome (4, 5).

The frequency of pathogenic MECP2 mutations among male individuals with intellectual disability (ID) is ~2% (6). While there are some variations in describing the male MECP2 phenotypes (7, 8), most three broader phenotype groups are differentiated. In the OMIM database, it is described as (1) severe neonatal encephalopathy (MIM:300673), (2) X-linked intellectual developmental disorder 13 (also known as pyramidal signs, parkinsonism, macroorchidism, and PPM-X syndrome) (MIM:300055), and (3) X-linked syndromic intellectual developmental disorder, Lubs type (MIM:300260). The duplication of the MECP2 gene causes this latter type. The GeneReviews database (2) also lists the neonatal encephalopathy group and PPM-X syndrome and lists syndromic/non-syndromic intellectual disability as a third group and MECP2 duplication as a separate entity (9).

There are some known genotype–phenotype correlations in male individuals affected by MECP2 disorders (10). In the severe neonatal encephalopathy group, patients harbor typical Rett syndrome-causing variants, but somatic mosaicism or XXY sex chromosomal pattern in the Klinefelter syndrome can mitigate the severe phenotype. The MECP2 duplication syndrome is usually associated with severe intellectual disability and neurological comorbidity. Patients in the syndromic and non-syndromic intellectual disability groups carry different variants than the typical Rett syndrome-causing variants. This latter group can also carry missense variants or late truncating variants in the C-terminal region or missense variants in the MBD/TRD connecting peptide (11).

The exact function of the MeCP2 protein and how its defect results in human disease is not fully understood, but many advances have been made since the description of the gene in the background of Rett syndrome (1). MeCP2 is ubiquitously expressed but has a predominant expression in neurons (12). Even though MeCP2 is an intrinsically disordered protein, there are well-defined domains in it: the N-terminal (NTD), the methyl binding (MBD), the intervening (ID), the transcription repression (TRD), the NCoR interaction (NID), and the C-terminal domains (CTD) (13). The current view is that the main action of the MeCP2 protein is related to transcriptional repression through the binding of its MBD to target gene promoters and recruiting histone deacetylases and co-repressors through the TRD. In this model, the MeCP2 serves as a bridge between methylated DNA and its main interactor, the NCoR/SMRT (nuclear receptor co-repressor/silencing mediator of retinoic acid and thyroid hormone receptor) co-repressor complex. In addition to this, there is a plethora of evidence that it can also act as an activator of gene expression through CREB1 recruitment or FOXO3 deacetylation by interacting with HDAC3 (14, 15). MeCP2 has also proposed post-transcriptional regulator effect, miRNA processing modification effect, and genome-wide effects on chromatin architecture and histone modifications (15, 16). These widespread changes in the neural epigenome alter diverse biological processes such as lipid metabolism, immune response, mitochondrial function, synaptic plasticity, neuronal functioning, and maintenance (16).

As MeCP2 is a broad regulator of gene expression, it is known to control the expression of the mitochondrial genome and nuclear mitochondrial genes as well (17, 18). Since early publications at the end of the 1980s, before the knowledge of the exact genetic background, clinical resemblance to mitochondrial disease was already appreciated (19), and ancillary studies also pointed to abnormal mitochondria (20). Since then, much information has been gathered about this aspect of the disease pathomechanism (17). On the other hand, in the clinical setting, especially if not presenting with a typical Rett phenotype, some patients with an MECP2 variant might be investigated for a suspected mitochondrial disease. In this article, we report a patient who showed atypical neuropsychiatric symptoms and evidence of mitochondrial encephalomyopathy associated with a pathogenic MECP2 rare variant. This patient showed mitochondrial dysfunction on different biological levels (transcriptome, muscle biopsy, and ergometric lactate test).

## Patients and methods

The study summarizes the diagnostic work-up and long-term follow-up of a 34-year-old man. Based on the diagnostic results, we conducted further studies to understand the effect of the MECP2 gene alteration and its therapeutic implications. The study was conducted according to the Declaration of Helsinki. The patient signed a written informed consent for publication.

A board-certified neurologist conducted a neurological examination. Routine laboratory testing, endocrinological assessment, EEG, and brain MRI were performed. Neuropsychiatric testing included the Addenbrooke's Cognitive Examination (ACE Hungarian version), as part of this, the Mini-mental State Examination (MMSE), Beck Depression Inventory (BDI), Beck Anxiety Inventory (BAI), Positive and Negative Syndrome Scale (PANSS), Montreal Cognitive Assessment (MOCA), and the Revised Autism Diagnostic Interview (ADI-R) were added. Extrapyramidal adverse effects were assessed with the Barnes Akathisia Rating Scale (BARS), Abnormal Involuntary Movement Scale (AIMS), and Simpson-Angus Scale (SAS).

An ergometric lactate stress test was performed to evaluate the mitochondrial function. In this test, the patient has to achieve maximal capacity cycling for 20 min. We draw blood for lactate measurements without a tourniquet before the test and immediately after it, and then after 5, 15, and 30 min after finishing cycling. This lactate stress, based on literature (21–23) and our own experience, is a good screening test to identify mitochondrial dysfunction. If the 30-min serum lactate level is at least twice as high as the resting level, the test indicates an aerobic metabolism disturbance.

Using the clinical, biochemical, and imaging data, we applied the Mitochondrial Disease Criteria score system (MDC score), a widely used diagnostic scoring system (24). A score of 1 indicates unlikely mitochondrial disorder, a score of 2–4 possible mitochondrial disorder, a score of 5–7 probable mitochondrial disorder, and a score of 8, which is the maximum, definite mitochondrial disorder.

To visualize the mitochondrial alterations, we performed a muscle biopsy from the right deltoid muscle under local anesthesia by a surgeon. Muscle biopsy specimens were frozen and stained with hematoxylin–eosin, ATPase on different pHs, PAS, Sudan Black B, and acidic phosphatase. To assess oxidative phosphorylation, we performed modified Gomori trichrome, modified SDH, and NADH-TR staining [the presence of ragged red fibers with modified Gomori trichrome staining, or ragged blue fibers on the modified SDH staining, might indicate abnormal mitochondrial aggregation. There is no strict cutoff widely accepted for the number of normally appearing ragged red (or raged blue) fibers, but 1–2% ragged red fibers in patients between 30 and 50 years can be considered abnormal (25)]. The ultrastructural abnormalities were detected by electron microscopy.

For genetic testing, we isolated DNA from the peripheral blood sample and performed short-read whole-exome sequencing on the Illumina platform. We filtered variants in the patient with the following criteria: (1) Known pathogenic/likely pathogenic variants in the ClinVar database. (2) All variants present in the mitochondrial genome. (3) Variant with minor allele frequencies of <1%, the variant is in exon ±10 bp, and variant is non-synonymous in virtual gene panels for nuclear mitochondrial genes; Mendelian diseases with psychiatric symptoms. (4) All variants classified by Franklin Genoox software as pathogenic or likely pathogenic. (5) Variants with a minor allele frequency of <0.001, the variant is in exon ±2, and the gene is on OMIM morbid map list and is a stop/frameshift/canonical splice site/deleterious missense variant. (6) Compound heterozygous or homozygous variants with MAF < 5% and non-synonymous variants. (7) Automatically prioritized variants of Franklin Genoox software. Variants were classified according to ACMG guidelines (16).

We performed RNA sequencing to study the functional consequences of the MECP2 variant in the patient. Peripheral blood was collected in PAXgene tubes. RNA isolation from whole blood and sequencing on the Illumina platform was performed by Novogene company. We compared the expression data from the patient in two time points and ten age-matched control samples. Reads were mapped to the Human GRCh38.p13 reference genome. Differential gene expression analysis was done by the Novogene Company. The bioinformatics analysis contained mapping to the reference genome with HISAT2, lncRNA identification with StringTie software, quantification with StringTie-eB, differential expression analysis with gallblown/Cuffdiff, and enrichment analysis. The enrichment analysis simply compared genes annotated to one pathway per all differentially expressed genes to the proportion of genes annotated to that pathway in the background gene sets.

## Results

### Patient description

The perinatal history of the patient was without any complications. During the first months, he was examined for failure to thrive, but examinations did not reveal any pathology. He had delayed speech development. For the length difference in the lower limbs (1 cm) and scoliosis since the age of 17 years, he was followed by an orthopedist. During his early school years, he was a cheerful, talkative child, but from the age of 10 years, he showed increasing social withdrawal and specific learning disabilities (dysgraphia, dyscalculia, and dyslexia). At the age of 17 years, a Wechsler intelligence test measured a low average IQ (total score 85; VQ: 94; PQ: 78). At that time, a psychiatric examination revealed anxiety and depression. A second test was performed at the age of 24 years and measured an IQ of 89 (VQ: 100; PQ: 80).

Family history: The proband's mother has had short stature, hypoacusis, and anxiety from age 7 years. His maternal grandmother also had a short stature. His brother had delayed psychomotor development in his early childhood. Presently, he and his son are also healthy.

At the first investigation in our Institute, the patient was 34 years old and had severe anxiety, panic attacks, somatic complaints, and exercise intolerance. He was able to leave the house only with his parents and had no social relationships outside his family. At that time, he was taking escitalopram 10 mg and clobazam 10 mg bid, but this regime did not control his symptoms. During the first exploration, we noticed severe anxiety, apathy, amotivation, avolition, alogia, and cognitive slowing; he was almost speechless. Physical examination did not detect any focal neurological signs, but we noticed microcephaly, micrognathia, mild arm rigidity, generalized myohypotrophy, and short stature. Macroorchidism was not present; instead, smaller testes were observed. Psychological examination revealed severe anxiety, dysthymia, anhedonia, and avolition. The results of the neuropsychological test are summarized in **Table 2**. Because of childhood history and restrictive social interactions, we raised the possibility of autism spectrum disorder. On the Revised Autism Diagnostic Interview (ADI-R), the total score was 40. In detail, the social domain scored 15 (cutoff 10), the communication and language domain scored 23 (cutoff 8), and the restricted interest and stereotype domain scored 2 (cutoff 3).

Based on the clinical history, we suspected mitochondrial disease, supported also by the MDC score (24). Scores and details on the clinical symptoms are presented in Table 1.

**Table 1:** Scores of the patient on the Mitochondrial Disease Criteria with an explanation of the positive findings.

**Mitochondrial Disease Criteria**		**Score**	**Comment**
Muscular	Myopathy	1	Fatigue, mild weakness, muscle atrophy, myopathological changes in biopsy
Max. score is 2	Abnormal EMG	0	
	Motor developmental delay	0	
	Exercise intolerance	1	
Neurological	Developmental delay or ID	1	Learning difficulties
Max. score is 2	Speech delay	1	
	Dystonia	0	
	Ataxia	0	
	Spasticity	0	
	Neuropathy	0	
	Seizures or encephalopathy	0	
Multisystem	Gastrointestinal tract disease	0	
Max. score is 3	Growth delay or failure to thrive	1	Failure to thrive, growth difference in the lower limbs
	Endocrine	1	Testosterone deficiency
	Immune	0	
	Eye (vision) or Hearing	0	Tinnitus
	Renal tubular acidosis	0	
	Cardiomyopathy	0	
Total maximal clinical score is 4		1 + 2+2 reaches total score: 4	
Metabolic	Lactate high at least 2x (score:2)	2	
	Alanine high at least 2x	n/a	Not measured
	Krebs cycle intermediates	n/a	Not measured
	Ethylmalonic and methylmalonic acid	n/a	Not measured
	3 methyl glutaconic acid	n/a	Not measured
	CSF lactate, alanine	n/a	Not measured
Imaging and other	Leigh disease (score: 2)	0	
	Stroke-like episodes (score:2)	0	
	Lactate peak on MRI	0	
	Leukoencephalopathy with brainstem and spinal cord involvement	0	
	Cavitating leukoencephalopathy	0	
	Leukoencephalopathy with thalamus involvement	0	
	Deep cerebral white matter involvement and corpus callosum agenesis	0	
Total maximal metabolic and MRI score is 4		Total metabolic and MRI score: 2	
Total MDC score (clinical, metabolic, imaging)		Total MDC score is 6 (probable mitochondrial disorder)	
Total score is maximal 8			

The table indicates the subscales and the assessed scores of the MDC. MDC, Mitochondrial Disease Criteria. A score of 1 indicates unlikely mitochondrial disorder, a score of 2–4 indicates possible mitochondrial disorder, and a score of 5–7 indicates probable mitochondrial disorder. A total score of 8 is a definite mitochondrial disorder.

### Detailed results of the laboratory investigations

**Figure 1:**
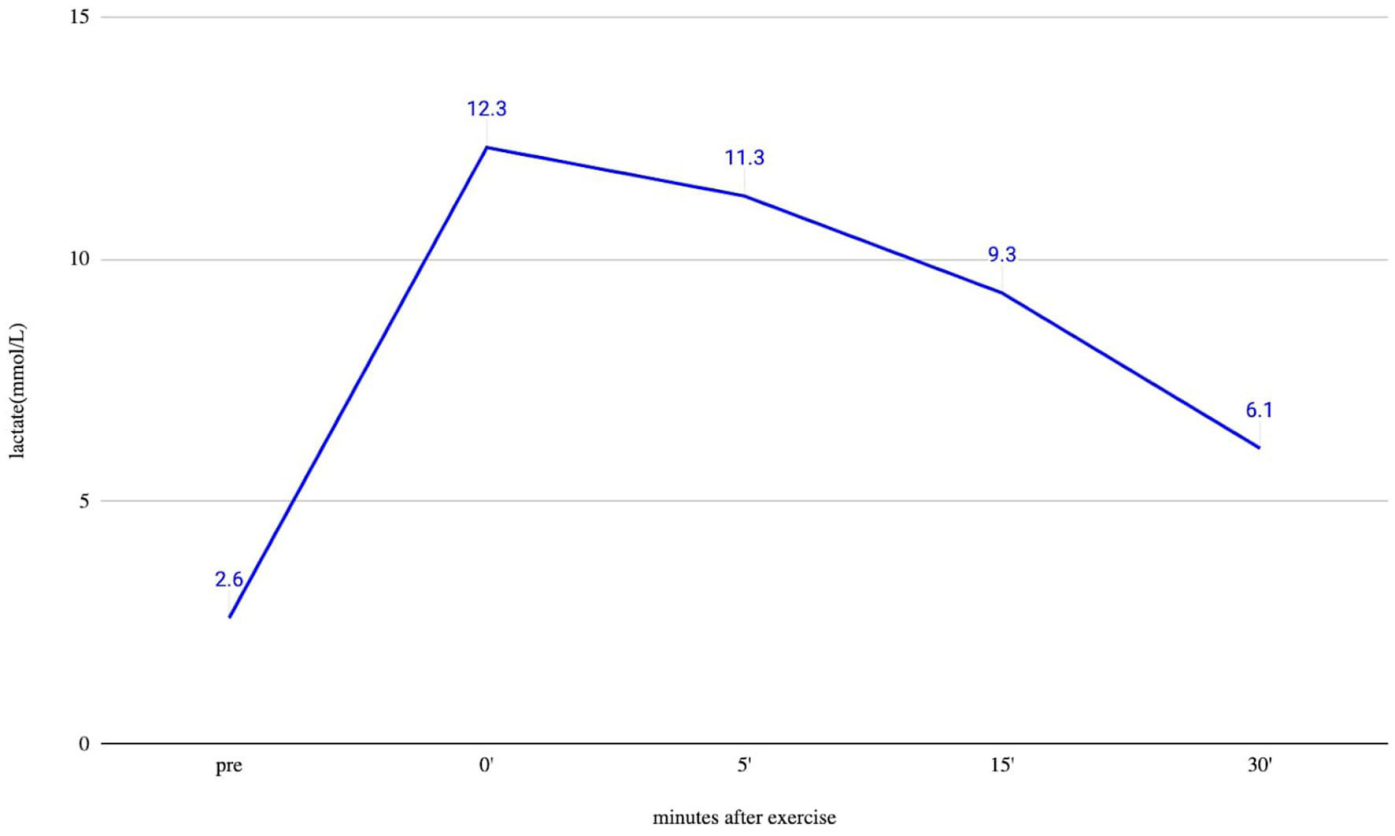
Ergometric lactate stress results of the patient. Lactate from peripheral venous blood was taken before exercise (pre) and immediately after finishing exercise (0), and after that at 5, 15, and 30 min. The measured resting lactate levels were normal. During an ergometric stress test, the lactate level steeply increased, and 30 min after the stress, the lactate level was still higher than double the resting level, which supports the disturbed function of muscle metabolism.

**Figure 2:**
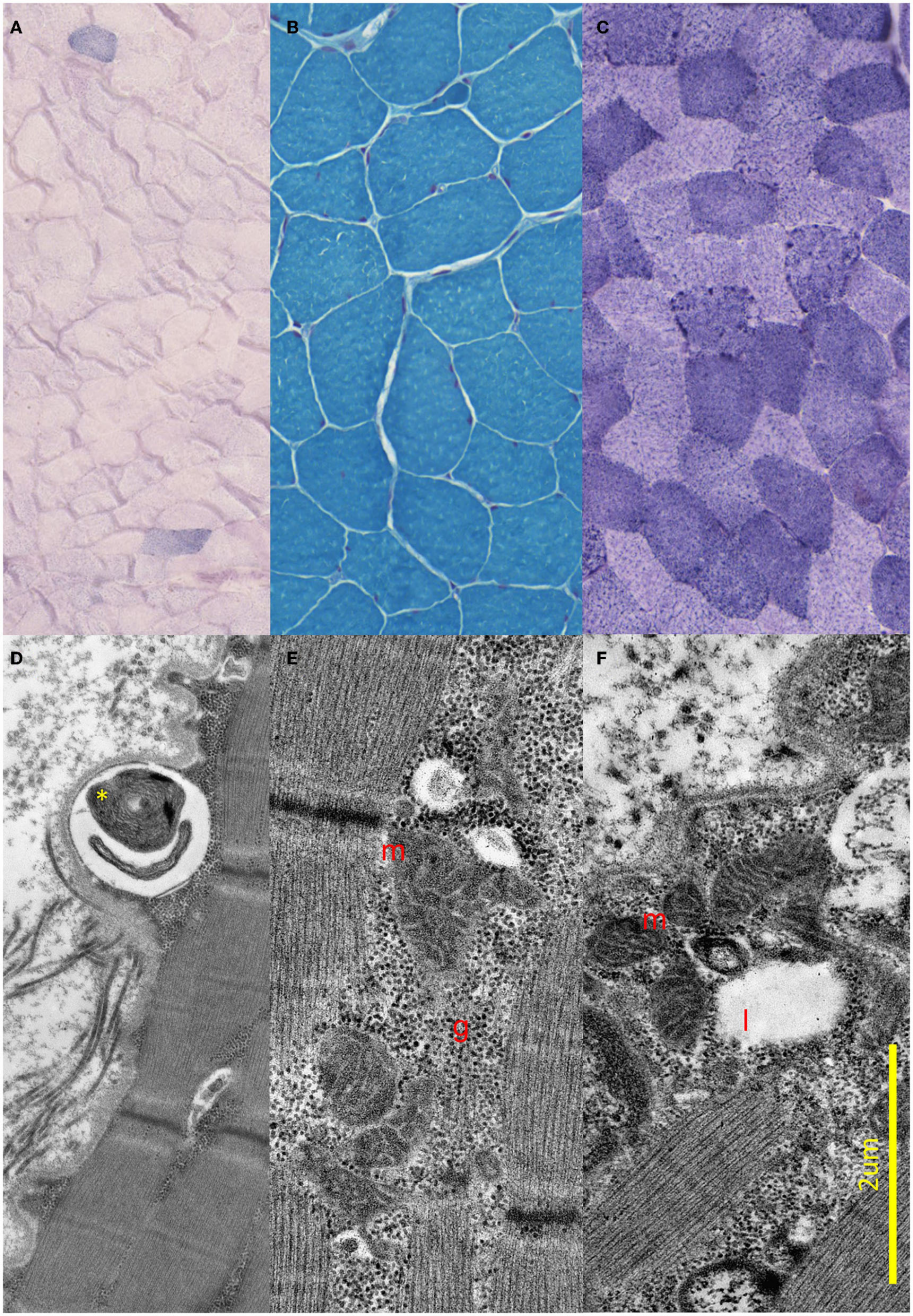
Light and electron microscopic results of the muscle biopsy. **(A–C)** Light microscopic images of the proband. **(A)** Modified SDH images showing two ragged blue fibers. **(B)** Gomori trichrome staining shows small angular, partially atrophic, and fewer significantly atrophic fibers consistent with a neurogenic lesion. **(C)** Fibers stained unevenly with NADH; in some fibers, subsarcolemmal increased Enzyme activity was present. **(D–F)** Electron Microscopic images of the proband. **(D)** Demonstrates concentric degenerated mitochondria (yellow star) in subsarcolemmal localization. **(E)** Shows intermyofibrillar, and **(F)** Shows subsarcolemmal increased number of enlarged, pleomorphic mitochondria (red m), glycogen accumulation (red g), and few lipid vacuoles (red l).

Brain MRI and EEG were normal. The serum total and free testosterone levels were decreased, and the prolactin level was elevated. The ergometric lactate stress test (Figure 1) revealed aerobic metabolism disturbance. In the muscle biopsy specimen (Figure 2), taken from the right deltoid muscle, the muscle fiber caliber variability was slightly increased, scattered rounded atrophic fibers were present, and a few angular atrophic fibers were detected occasionally. Three ragged blue fibers were identified by modified SDH staining. In some muscle fibers, subsarcolemmal increased cytochrome oxidase C (COX) activity was detected, which was present by NADH-TR staining, as well. Electron microscopy revealed intermyofibrillar and subsarcolemmal enlarged mitochondria. Frequently, lipid vacuoles were observed in the vicinity of the degenerated mitochondria. Some mitochondria had either degenerated or abnormal concentric cristae. Intermyofibrillar mild glycogen accumulation was also present.

The whole-exome sequencing detected a hemizygous likely pathogenic missense variant in the MECP2 gene: NM_001110792.1(MECP2):c.535C>T (p.Arg179Trp). This variant was already present in the ClinVar database under ID:143603 with the following classifications: likely pathogenic (1); pathogenic (4); uncertain significance (1). The variant is missing from the large population databases and is located in a mutational hotspot. Based on the available information, we classified the variant as likely pathogenic. No pathogenic or likely pathogenic variants were detected in the mitochondrial genome. In the nuclear mitochondrial genes, only heterozygous variants of uncertain significance were detected in autosomal recessive genes. We performed a segregation analysis for the MECP2 variant in the family. The variant was heterozygous in the mother and absent in his brother.

**Figure 3:**
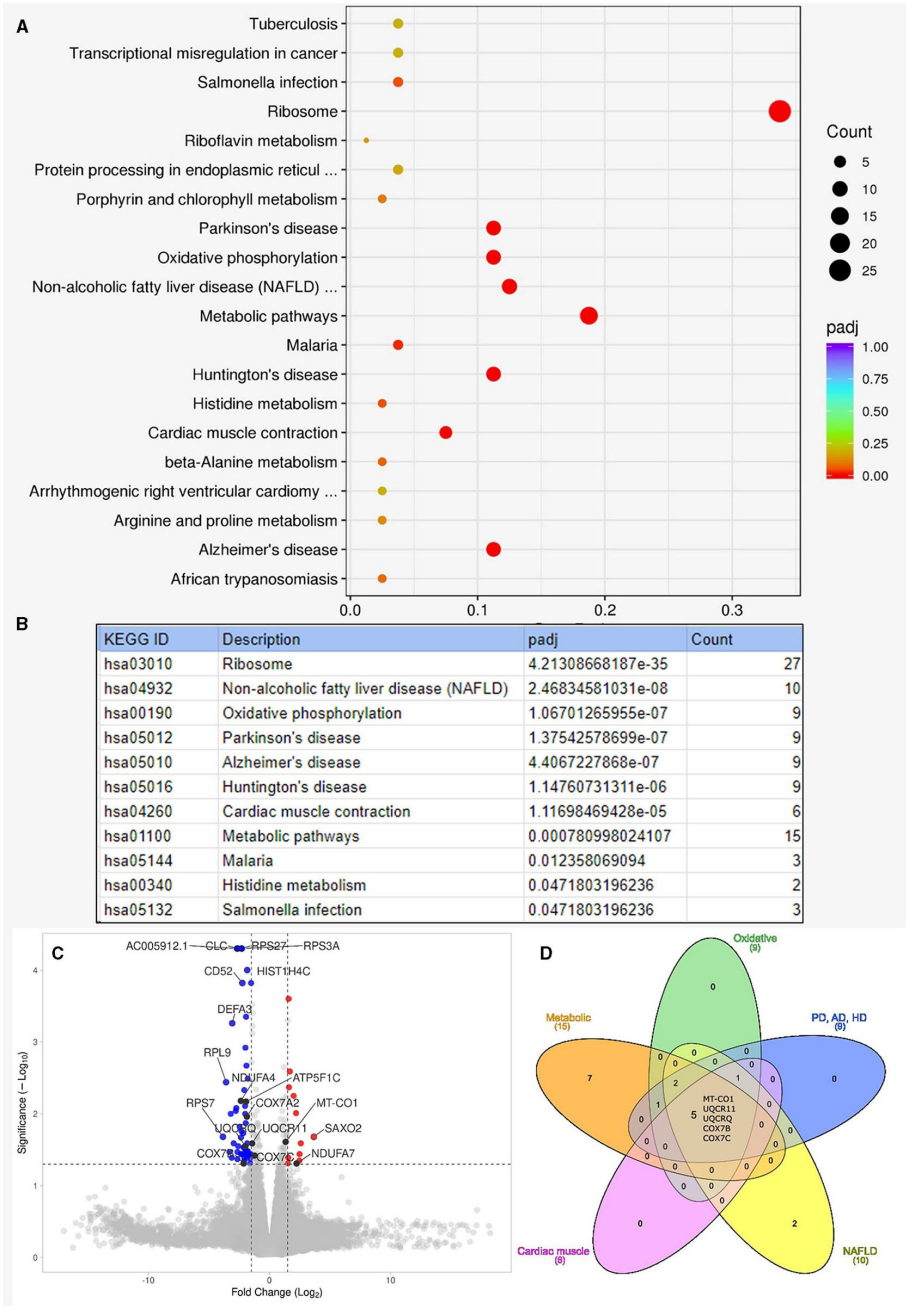
Results of enrichment analysis. **(A)** Shows the summary result of the enrichment analysis. On this enrichment scatter plot, the y-axis shows the pathway's name, and the x-axis shows the rich factor. Color represents the adjusted *p*-value and the size of the circle of the gene count. Rich factors refer to the ratio of DEGs number in the pathway and the number of all genes annotated in the pathway. **(B)** Lists the pathways that showed significant enrichment among the Kyoto Encyclopedia of Genes and Genomes (KEGG) pathways with adjusted *p*-values (padj). “Count” is the number of genes detected to be differentially expressed on the given pathway. **(C)** Shows the volcano plot for the enrichment analysis. Altogether, 162 genes were differentially expressed: 54 upregulated and 108 downregulated with moderate log-fold changes. Dashed lines indicate the thresholds for fold change (−1.5, 1.5) and -log10p-value (1.3). Red dots are upregulated, and blue dots are downregulated genes. We indicated the top 10 genes according to Manhattan distance, plus the 9 mitochondrial genes with black dots. A volcano plot was generated with the VolcaNoseR (26). **(D)** Shows the overlapping differentially expressed genes among different pathways. The Venn diagram was generated with the InteractiVenn (27). AD, Alzheimer's disease; HD, Huntington's disease; NAFLD, non-alcoholic fatty liver disease; PD, Parkinson's disease.

In the RNA overrepresentation study, differentially expressed genes were first ordered according to nominal *p-*values. Selecting a cutoff of p≤0.05 for nominal *p*-values, altogether 54 genes were upregulated, and 108 genes were downregulated (Supplementary Table 1). While alone no single gene showed significant differential expression when adjusting *p*-values for multiple hypothesis testing, in the enrichment analysis, we have seen that the oxidative phosphorylation pathways showed significant enrichment in addition to ribosomes, non-alcoholic fatty liver disease, and neurodegenerative disease pathways. When looking at the individual genes in the above pathways, it turned out that one prominent group of genes were ribosomal protein-coding genes. The genes on the oxidative phosphorylation, metabolic, Parkinson's disease, Alzheimer's disease, Huntington's disease, non-alcoholic fatty liver disease, and cardiac muscle contraction pathways overlapped considerably. The genes on these pathways were coding, to a large extent, mitochondrial proteins. The five genes present in all the above pathways are all mitochondrial oxidative phosphorylation-related genes: MT-CO1, UQCR11, UQCRQ, COX7B, and COX7C. The results of the KEGG overrepresentation enrichment analysis method by Novogene are shown in Figure 3.

### Treatment approaches

Since the treatment with SSRI and benzodiazepine was not effective, and the patient had severe negative psychiatric symptoms, we initiated cariprazine 1.5 mg. Four months later, the cariprazine dose was increased to 3 mg since the 1.5 mg had only a mild positive effect, and procyclidine was administered since a resting tremor had occurred. Six months after starting cariprazine, the symptoms improved, but he was still socially severely disabled, so the dose was increased to 4.5 mg. This dose improved the negative and affective symptoms (see the scores in Table 2), but at this point, moderate bradykinesia and rigidity appeared, so we decreased the cariprazine dose to 3 mg and increased the procyclidine dose to 3 × 5 mg. Since the extrapyramidal side effect of the cariprazine was not tolerated, we added a higher dose of venlafaxine to the therapy instead of escitalopram. He also received a mitochondrial cocktail with coenzyme Q10, folic acid, C-vitamin, B2 vitamin, and B3 vitamin. The medications and the change in the psychological rating scale and movement scales are presented in Table 2.

**Table 2:** Treatment regime and its effect on the neuropsychological scores.

**Date**	**06/2020**	**02/2021**	**04/2021**	**06/2021**	**07/2021**
Escitalopram	1 × 10 mg	1 × 10 mg	1 × 10 mg	1 × 10 mg	1 × 10 mg → 0 mg
Venlafaxine	-	-	-	-	0 mg → 75 mg
Clobazam	2 × 10 mg	2 × 10 mg	2 × 10 mg	2 × 10 mg	2 × 10 mg
Procyclidine	-	0 mg → 2 × 5 mg	2 × 5 mg	2 × 5 mg → 3 × 5 mg	3 × 5 mg
Cariprazine dose	0 mg → 3 mg	3 mg → 4.5 mg	4.5 mg	4.5 mg → 3 mg	3 mg
ACE	-	87	89	92	96
BDI	17	12	3	7	9
BAI	35	30	19	18	21
PANSS	-	34	33	30	28
AIMS	-	0	0	0	0
BARS	-	0	0	0	0
SAS	-	6	6	7	7
MOCA	19/30	-	-	-	-
MMSE	-	26/30	28/30	28/30	28/30

The table shows the treatment regime and scores on different psychological rating scales and movement scales. The first time point (06/2020) is retrospective; thus, certain scores are missing. Arrows indicate treatment changes at the visit. ACE, Addenbrooke Cognitive Examination; BDI, Beck Depression Inventory; BAI, Beck Anxiety Inventory; PANSS, Positive and Negative Syndrome Scale; AIMS, Abnormal Involuntary Movement Scale; BARS, Barnes Akathisia Rating Scale; SAS, Simpson-Angus Scale; MOCA, Montreal Cognitive Assessment; MMSE, Mini-Mental State Examination.

## Discussion

We presented for the first time a male patient carrying a pathogenic MECP2 p.Arg179Trp variant with negative symptoms and autistic-like features responding well to the cariprazine treatment. He had delayed speech development and later experienced severe social anxiety, learning disabilities, cognitive slowing, and persistent negative symptoms (alogia, apathy, amotivation, and avolition). In addition to the neuropsychiatric symptoms, upper limb rigidity, muscle hypotrophy, and exercise intolerance were seen. We suspected mitochondrial disease. The muscle biopsy, ergometric lactate test, and RNA sequencing provided evidence for the mitochondrial dysfunction.

Our case highlights the diagnostic challenges in male MECP2-associated disorders due to the variability in clinical presentation, lack of specific diagnostic criteria, and non-specific laboratory findings such as brain imaging or EEG. Difficulties in the differential diagnosis of male MECP2-associated phenotypes are further complicated by symptoms that have not been previously reported, such as persistent negative symptoms.

Negative symptoms can be a clinical sign of dopamine imbalance. Further signs of the altered dopamine dysfunction in Rett syndrome are the Parkinsonian features, such as hypomimia and rigidity, bradykinesia, and cognitive impairment (28). In Mecp2-null mice, reduction in dopamine content in the striatum explains motor deficits (29). The MECP2-associated disorders, including Rett syndrome, share some similarities with autism spectrum disorders, and dopamine dysregulation may also contribute to the development of autistic-like behaviors (30). There are many potential therapeutic options for Rett syndrome targeting either the genetic origin of the disease or its downstream consequences, reviewed extensively by N. Vashi and MJ. Justice (31). One important area is neurotransmitter signaling, including dopaminergic pathways.

Cariprazine is a partial agonist at the D2/D3 dopamine receptors, revealing the highest *in vivo* D3R affinity among all molecules approved for treating psychiatric disorders and a higher affinity for D3R than dopamine at clinically relevant doses. In addition to this, cariprazine acts on the serotonin 5-HT1A receptors and 5-HT2B receptors, as well (32). Its clinical consequences are improvements in negative, cognitive, and affective symptoms, as well as in motivation and reward (33). In different neurological and psychiatric disorders with predominant negative psychiatric symptoms such as schizophrenia or Huntington's disease, among others, in addition to the involvement of the mesolimbic and mesocortical dopaminergic pathways, the frontostriatal pathway is also affected. Cariprazine has the potential to improve cognitive and mood symptoms in these cases (34, 35). In Rett syndrome, mainly the frontal cortex is altered, so we assume that the partial agonist effect on the D3 receptor may have a positive effect on the negative psychiatric symptoms in Rett syndrome as well. Cariprazine is the only antipsychotic with proven superiority over other antipsychotics in the treatment of persistent negative symptoms, including anhedonia, avolition–apathy, and alogia (36). Previously, we reported positive observations about cariprazine treatment in mitochondrial encephalomyopathy and lactic acidosis (MELAS) syndrome due to the mutation m.A3243G, where the predominantly negative symptoms and cognitive dysfunction improved (37). The above observation is important clinically as it is known that many antipsychotics inhibit the oxidative phosphorylation processes and have the potential to worsen the primary mitochondrial dysfunction (38, 39).

The detected disease-causing p.Arg167Trp variant in the MECP2 variant in our patient is present in the literature (Table 3). Rs61748420 is a C>T change at codon 167, resulting in an Arg-Trp substitution (R167W, p.Arg167Trp) in MECP2. This missense variant is between the methyl-CpG-binding domain (MBD) and transcription repression domain (TRD), so the relevance of the mutation is more challenging to describe (41). With the presence of this variant, a significant increase in the number and size of chromocenters and heterochromatin clustering defects was observed. DNA-binding dynamics of MeCP2 depend on the amino acid structure of MBD. The impaired molecular behavior of MBD is highly significant for developing the clinical picture, which varies on a broad spectrum between severe and mild phenotypes (40). In summary, MECP2-related disease in males represents a broad spectrum. While most reports of the p.Arg167Trp variant mention intellectual disability, the patient we have studied had average intelligence, but severe psychiatric symptoms dominated the clinical picture. Our observation supports that markedly different clinical pictures can be present even with the same variant in the MECP2 gene.

**Table 3:** Patients carrying MECP2 p.Arg167Trp from the literature.

**Study**	**Main clinical feature**	**Inheritance**	**Additional symptoms**
Neul et al. (3)	One man with cognitive impairment	Inherited from an asymptomatic mother	Hand clapping, finger rubbing lost but later regained hand skills, peripheral vasomotor symptoms, scoliosis, bruxism, sleep issues, screaming, and poor pain response
Bianciardi et al. (11)	Three affected brothers with language delay and learning disabilities	Inherited from an asymptomatic mother	
	Patient 1: severe intellectual disability		Apathy, anxiety, hypochondria, shyness, obsessive demand for food, hypersomnia, short stature, obesity, macrocephaly, downturned nasal tip with wide pinnae, short philtrum, anteverted ears, and large hands
	Patient 2: moderate intellectual disability		Poor language, aggressiveness, low frustration tolerance, episodes of inappropriate laughter, apathy, obsessive demand for food, hypersomnia, short stature, obesity, high forehead, downturned nasal tip with wide pinnae, short philtrum, thin lips, posteriorly rotated ears, and large hands
	Patient 3: severe intellectual disability		Language limited to simple sentences, social impairments, unmotivated laughter, apathy, obsessive demand for food and hypersomnia, high myopia, keratoconus, short stature, obesity, macrocephaly, cleft right ear lobe, and similar facial appearance as in brothers
Sheikh et al. (40)	One man with developmental delay	No information	Mild motor delay and short phrases
Couvert et al. (6)	Four men with non-specific intellectual disability from one family	Inherited from asymptomatic mothers	
	Two carrier women had normal intelligence but non-progressive slight resting tremor		-
	Patient 1: mild to moderate intellectual disability		Seizures, brisk tendon reflexes, tremor, hypoactive, and sometimes agitated
	Patient 2: mild intellectual disability		Decreased occipitofrontal circumference, brisk tendon reflexes, tremor, clumsiness, and hypoactive
	Patient 3: mild intellectual disability		Decreased occipitofrontal circumference, language delay, tremor, and sometimes irritable
	Patient 4: mild intellectual disability		Decreased occipitofrontal circumference, seizures, brisk tendon reflexes, ataxia, anxiety, and emotional disturbance

We searched the literature with Mastermind Genomic Intelligence Platform (https://www.genomenon.com/mastermind) for NM_001110792.1(MECP2):c.535C>T (p.Arg179Trp) variant. We did not include in this table cases where the MECP2 p.Arg167Trp was detected alongside another variant in the MECP2 gene or another gene.

Evidence for mitochondrial dysfunction was described in early reports of Rett syndrome (42). For example, Dotti et al. (43) described the muscle biopsy of two women with Rett syndrome, where swollen and aberrant mitochondria were detected with concentric laminated bodies of filamentous material. In some Rett syndrome studies, increased lactate and pyruvate concentrations were registered in blood or cerebrospinal fluid, but it was also suggested that this could be a secondary phenomenon (20, 44, 45). Since then, many confirmed suspected mitochondrial dysfunction in patients with Rett syndrome on clinical (e.g., lactate elevation), structural, and transcriptomic levels. For a comprehensive review, we refer to the study by Shulyakova et al. (17). There is also evidence for redox alterations, increased oxidant burden, and oxidative damage (46). It is worth mentioning that in a larger mitochondrial patient cohort (47), one patient carried a frameshift variant in the MECP2 gene and was reported to present complex I deficiency, thus indicating, in addition to our case, that indeed some suspected mitochondrial patients have a pathogenic variant in the MECP2 gene.

As MeCP2 is a transcriptomic regulator, many studies tried to elucidate the pathomechanism through transcriptomic studies. Transcriptomic analysis from different tissues (brain sample, blood, and iPSC) suggested the involvement of many different pathways, which was reviewed by Shovlin and Tropea (48). In addition to abnormalities in dendritic arborization, synaptic maturation, glial cell activity pathways, and mitochondrial dysregulation were the main categories highlighted by these studies.

We used blood transcriptomics in our patients primarily for practical reasons. While it has obvious drawbacks, peripheral blood samples were used before for transcriptomic studies in Rett syndrome. For example, Pecorelli et al. described upregulation of genes with mitochondrial function (49). Colak et al. (50) found that patients with Rett syndrome-like phenotype showed transcriptional evidence of mitochondrial dysfunction but not patients with classic Rett syndrome. Blood-based quantification showed higher mtDNA copy numbers in a study with a cohort of patients with Rett syndrome. The authors hypothesized that this is a compensatory mechanism caused by impaired energy metabolism and increased oxidative stress (51).

While blood is not the most relevant tissue to study in Rett syndrome, there is also evidence of mitochondrial involvement from other tissues. In Rett syndrome, brain abnormalities are mainly found in the frontal cortex. Testing this region in postmortem brain tissue from patients with Rett syndrome, the following differentially expressed genes were detected: cytochrome c oxidase subunit 1, clusterin, and dynamin 1. In these samples, RNAi-mediated knockdown of MECP2 showed respiratory chain defect through cytochrome c oxidase subunit 1 (52). In most instances, mitochondrial genes were upregulated. However, in the frontal cortex, MT-CO1 was downregulated in patients with Rett syndrome (48, 52). A proteomic analysis of patient-derived fibroblasts with Rett syndrome proved the same abnormal mitochondrial function with the expression changes of proteins in mitochondrial structure, dynamics, function, related pathways, cellular stress defense, and mitophagy (53). In MECP2 knockdown astrocytes, decreased respiratory chain complex activities, mitochondrial membrane potential reduction, and intracellular calcium release were observed (53). A recent study by Zlatic et al. (54) expanded our knowledge with two interesting aspects regarding metabolic changes in Rett syndrome. Zlatic et al. (54) performed a simultaneous transcriptomic and proteomic analysis in multiple organs and brain regions from Mecp2-null mice and performed mitochondrial respiration studies. Their analysis showed that Mecp2-sensitive transcriptomes and proteomes might diverge, and enrichment in lipid metabolism and mitochondrial pathways is more prominent in the proteomic analysis. On the other hand, regarding mitochondrial respiration, a selective metabolic defect was shown for pyruvate utilization, causing defective metabolic flexibility. Pascual-Alonso et al. (55) used multi-omics from patient fibroblasts to identify common elements in Rett syndrome, Rett-like phenotype, and MECP2 duplication syndrome. Dysregulation of cytoskeletal organization, vesicular activity, translation, and mRNA processing were common elements. While most studies focused previously on changes in neurons in the central nervous system, it was recently also shown that astrocytes are affected significantly by mitochondrial alterations (56).

Our results were congruent with the above findings. In our single patient, the lactate stress test was positive, muscle biopsy showed mitochondrial changes, and RNASeq analysis also showed involvement of metabolic pathways and oxidative phosphorylation. While we cannot draw far-reaching conclusions from one sample, it is reassuring that mitochondrial pathway involvement was seen in the RNASeq data of this single patient in parallel with the other results. In this analysis, some neurodegenerative pathways with mitochondrial alteration (Parkinson's disease, Huntington's disease, and Alzheimer's disease), non-alcoholic fatty liver disease pathway, and ribosome pathway were also perturbed. However, disturbed mitochondrial metabolism is not the only metabolic complication known to be present in Rett syndrome. Dyslipidemia, elevated plasma leptin and adiponectin, gallbladder inflammation, and changes in neurometabolites and brain carbohydrate metabolism were also reported (5). We did not detect dyslipidemia or cholecystitis in our patient; however, these are not always present.

The involvement of the ribosomal pathway parallels the widespread perturbation of transcription and translation in Rett syndrome (57, 58). In our differential expression analysis among the 162 genes with a non-adjusted *p*-value of ≤ 0.05, 29 genes were associated with ribosomes (Supplementary Table 1). Among these, 19 genes were downregulated, and 10 genes were upregulated. The log-fold values were greater for the downregulated genes (11/19 genes with a log-fold value smaller than −2.0, while no upregulated genes reached a log-fold value ≥ 2). Altogether, downregulation on this pathway seems more important than upregulation.

This wide repression of ribosomal genes is in harmony with the literature. It was suggested already more than 20 years ago that MeCP2 functions as a global transcriptional repressor (59). The recent model suggests that MeCP2 acts in a more complex way (57). It acts as a global activator for abundantly expressed genes (such as mitochondrial genes) and as a transcriptional repressor for a smaller group of normally lowly expressed genes. In the former study of Yun Li et al. (57), a prominent group of genes affected in MECP2 mutant neurons were ribosomal proteins, which, combined with the reduced level of rRNAs, results in a global decrease in translational capacity. Reduced global translation was recently shown by DC. Rodrigues et al. (58) used parallel translating ribosome affinity purification sequencing (TRAP-seq) and RNA sequencing (RNA-seq) to study ribosomal engagement. Among other biological pathways, they observed a consistent reduction in ribosomal engagement of ribosomal proteins and translation initiation factors consistent with our findings.

## Conclusion

The diagnostic classification of male Rett syndrome, typical or atypical, is more complicated than we ever thought. It could be caused by germline or somatic mutations of MECP2 or even by sex chromosomal abnormalities. We presented a case with a MECP2 pathogenic mutation and signs of dopamine imbalance, such as persistent negative symptoms and autistic-like features responding well to cariprazine treatment. Related mitochondrial dysfunction was identified on multiple biological levels for the first time in a male MECP2 variant carrier. This study further raises the importance of the MECP2 gene in the mitochondrial pathways, which can open the gate for more personalized therapeutic interventions. Good cariprazine response highlights the role of characterizing the disease pathomechanism of a clinical symptom. This can help identify the optimal treatment strategy from a transdiagnostic perspective instead of a classical diagnostic category.

## Data availability statement

The datasets for this article are not publicly available due to concerns regarding participant/patient anonymity. Requests to access the datasets should be directed to the corresponding author.

## Ethics statement

The studies involving humans were approved by the Hungarian - National Institute of Pharmacy (EudraCT Number: 2021-001268-58). The studies were conducted in accordance with the local legislation and institutional requirements. The participants provided their written informed consent to participate in this study. Written informed consent was obtained from the individual(s) for the publication of any potentially identifiable images or data included in this article.

## Author contributions

PB: Conceptualization, Data curation, Investigation, Methodology, Supervision, Visualization, Writing—original draft, Writing—review & editing. AGe: Data curation, Formal analysis, Investigation, Methodology, Visualization, Writing—review & editing. MF: Data curation, Formal analysis, Investigation, Writing—review & editing. JS: Conceptualization, Investigation, Methodology, Writing—original draft, Writing—review & editing. AGa: Data curation, Formal analysis, Investigation, Methodology, Writing—review & editing. NV: Conceptualization, Methodology, Writing—review & editing. MM: Conceptualization, Funding acquisition, Investigation, Resources, Supervision, Writing—original draft, Writing—review & editing.
